# Is stem cell transplantation still needed for adult Philadelphia chromosome-positive acute lymphoblastic leukemia receiving tyrosine kinase inhibitors therapy?: A systematic review and meta-analysis

**DOI:** 10.1371/journal.pone.0253896

**Published:** 2021-06-28

**Authors:** Ben Ponvilawan, Smith Kungwankiattichai, Nipith Charoenngam, Weerapat Owattanapanich

**Affiliations:** 1 Department of Pharmacology, Faculty of Medicine Siriraj Hospital, Mahidol University, Bangkok, Thailand; 2 Division of Hematology, Department of Medicine, Faculty of Medicine Siriraj Hospital, Mahidol University, Bangkok, Thailand; 3 Department of Medicine, Faculty of Medicine Siriraj Hospital, Mahidol University, Bangkok, Thailand; Stanford University, UNITED STATES

## Abstract

**Background:**

Hematopoietic stem cell transplantation (HSCT) is the current mainstay treatment for Philadelphia chromosome-positive acute lymphoblastic leukemia (Ph^+^ ALL). However, tyrosine kinase inhibitors (TKI) also play a significant role in the treatment of these patients. We conducted this systematic review and meta-analysis to compare the efficacy of allogeneic (allo-) HSCT, autologous (auto-) HSCT, and chemotherapy (CMT) alone–all in combination with TKIs in adult Ph^+^ ALL patients.

**Materials and methods:**

This systematic review identified studies from the EMBASE and MEDLINE databases from inception to April 2021 using search terms related to “ALL” and “HSCT.” Eligible studies could be randomized controlled trials or cohort studies that included adult Ph^+^ ALL patients who received a TKI and either allo-HSCT, auto-HSCT, or CMT alone, and that reported the number of patients in each group for each of our primary outcomes of interest: overall survival (OS) or disease-free survival (DFS). Point estimates and associated 95% confidence intervals (CI) from each study were combined using the Hantel-Maenszel method.

**Results:**

After two rounds of review, 26 cohort studies were determined to be eligible for the meta-analysis. Adult Ph^+^ ALL patients who received HSCT had better survival outcomes than those who did not receive any HSCT (pooled odds ratio [OR] for OS of 1.61, 95%CI: 1.08–2.40; I^2^ = 59%, and for DFS of 3.23, 95%CI: 2.00–5.23; I^2^ = 62% for allo-HSCT; and, pooled OR for OS of 7.04, 95%CI: 1.97–25.15; I^2^ = 0%, and for DFS of 5.78, 95%CI: 1.04–32.19; I^2^ = 42% for auto-HSCT). Allo-HSCT recipients had comparable OS and DFS, but lower relapse rate compared to auto-HSCT recipients. Funnel plot generally demonstrated no presence of publication bias.

**Conclusions:**

This systematic review and meta-analysis demonstrated superior results of HSCT in Ph^+^ ALL patients compared to CMT alone. Moreover, auto-HSCT could be implemented with comparable survival outcomes to allo-HSCT in patients with no available donor or when haploidentical HSCT is not feasible.

## Introduction

Philadelphia chromosome-positive acute lymphoblastic leukemia (Ph^+^ ALL) is a subtype of ALL which harbors the reciprocal translocation between chromosome 9 and 22, t(9;22)(q34;q11), causing the hallmark *BCR-ABL1* fusion gene. It is the most common cytogenetic abnormality which comprises 15–40% of all adult ALL cases [1–4].

Ph^+^ ALL is among the most adverse subtypes of ALL with poor prognosis and frequent relapses. Before tyrosine kinase inhibitors (TKIs) were incorporated to the standard of care, Ph^+^ ALL patients had a 5-year overall survival rate of 25% compared to approximately 50% in standard-risk ALL patients [5]. Allogeneic (allo-) hematopoietic stem cell transplantation (HSCT) is also considered as the mainstay of Ph^+^ ALL treatment as it could improve the 5-year overall survival rate to 35–44% [3, 4, 6]. However, donor unavailability and non-tolerance to intensive regimens usually limit allo-HSCT procedures, forcing patients to receive other modes of treatment; autologous transplantation or chemotherapy (CMT) without transplantation were employed, albeit the lower response and higher relapse rate [7].

The emergence of TKIs has remarkably shaped the treatment landscape of Ph^+^ ALL by improving the response rate and survival outcomes compared to historical cohorts without TKIs [8, 9]. Interestingly, there is growing evidence that suggests the non-inferiority of autologous (auto-) HSCT, or even CMT alone, compared to allo-HSCT in the era of TKIs although the published results are still conflicting [10–14].

Thus, we conducted this systematic review and meta-analysis to compare the survival outcomes and relapse rate of adult Ph^+^ ALL patients among three therapeutic strategies: allo-HSCT, auto-HSCT and CMT alone, in combination with TKIs.

## Materials and methods

### Data sources and searches

All relevant studies indexed in EMBASE and MEDLINE databases from inception to April 2021 were independently searched by three investigators (B.P., S.K., N.C.) using search terms associated with “ALL” and “stem cell transplantation”. The comprehensive list of search strategy used in this study is shown in S1 File. The systematic review and meta-analysis were performed in accordance with the Preferred Reporting Items for Systematic Reviews and Meta-analysis (PRISMA) guidelines as described in S1 Table.

### Selection criteria

The eligible study must be either cohort studies (either prospective or retrospective) or randomized control studies which had at least two groups of adult Ph^+^ ALL patients with at least 80% of patients receiving a TKI (any of imatinib, nilotinib, dasatinib, bosutinib, or ponatinib) during treatment. Each group also had to receive the same treatment of either allo-HSCT, auto-HSCT, or CMT alone without any transplantation (CMT). The study must report our primary outcome of interest: overall survival (OS) or disease-free survival (DFS). Secondary outcomes of interest consist of cumulative incidence of transplant-related mortality (TRM) and cumulative incidence of relapse (CIR), which will be collected if they are present in the study. All outcomes must be reported as the number of patients in each group to be eligible for the analysis. Study eligibility was separately determined by two investigators (B.P., S.K.). In case of different opinions, a consensus was established after discussion with the senior investigator (W.O.).

### Data extraction

Data from each study was extracted using a standardized data collection form which consists the following information: the first author’s surname, publication year, type of study, study period, type of transplantation treatment (allogeneic, autologous or CMT only), number of participants in each group, number of male and female participants in each group, median age and range of participants in each group, type and dosage of TKI, chemotherapeutic regimen, type of conditioning regimen (myeloablative conditioning, reduced-intensity conditioning or non-myeloablative conditioning) and donor type, in case of allo-HSCT (matched sibling, unrelated, haploidentical or umbilical cord blood).

### Definitions of outcomes

OS rate was defined as the ratio of patients who were still alive since the diagnosis date at a particular time of interest [14–16]. DFS and CIR rates were defined as the proportion of patients who did not have a relapse or death and the ratio of patients who had a relapse, after complete remission at the time of interest, respectively [12, 14]. TRM rate was defined as the percentage of patients who have had a recurrence or had died since the initiation date of transplantation (in case of patients in allo-HSCT and auto-HSCT groups) or treatment (in case of patients in CMT group) to a specific time of interest [14, 17]. For each outcome of the study, the longest duration in which the results are available was chosen as the time of interest.

### Quality assessment

The Newcastle-Ottawa quality assessment scoring system for cohort studies, which determines the study quality using 8-item criteria based on selection, comparability, and outcome of each cohort group, and the Jadad quality assessment scoring system for randomized controlled studies were used for the evaluation of the quality of each study by two investigators (S.K., W.O.) [18, 19].

### Statistical analysis

All statistical analyses were performed in Review Manager 5.3 software (The Cochrane Collaboration, United Kingdom). Effect estimates along with their 95% confidence interval (CI) were extracted from each study and combined to calculate the pooled odds ratio using the Mantel-Haenszel method [20]. As a result of the higher chance of interstudy heterogeneity, a random-effects model, in preference to the fixed effects model, was utilized in this study. Cochran’s Q test, together with the I^2^ statistic, was used to measure statistical heterogeneity. The I^2^ statistic numerically evaluates the proportion of the total variation across studies which is accounted by study heterogeneity rather than random chance, with the I^2^ value of 0–25% representing insignificant heterogeneity, 26–50% low heterogeneity, 51–75% moderate heterogeneity and >75% high heterogeneity [21]. A funnel plot was used for the determination of the presence of publication bias if the meta-analysis had an adequate amount of eligible studies. Subgroup analyses were also performed if there are sufficient amount of studies with all Ph^+^ ALL patients receiving TKIs, studies with all Ph^+^ ALL patients receiving post-transplant TKI, studies with all Ph^+^ ALL patients achieving first complete remission (CR1) before HSCT or CMT, and subgroup analysis stratified by the type of TKI used in the studies.

## Results

### Search results

**Fig 1:**
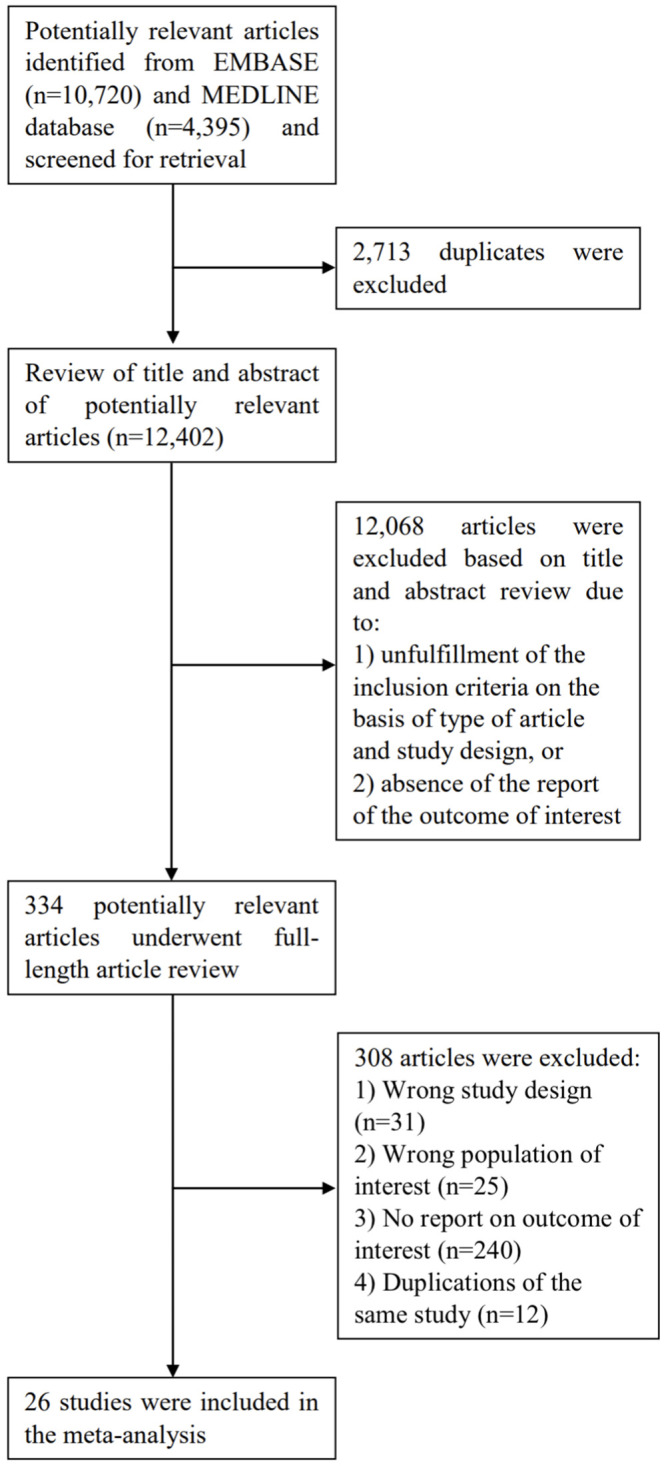
Study identification and literature review process.

A total of 15,115 articles were retrieved from a systematic search in EMBASE and MEDLINE databases in which 2,713 articles were duplications and removed, leaving 12,402 articles for title and abstract review. A round of title and abstract review discarded 12,068 as their article type and study design clearly did not satisfy the inclusion criteria. This resulted in 334 articles for full-length article review. A total of 308 articles were further excluded as they did not meet the inclusion criteria and report our outcomes of interest, ultimately leaving 26 eligible studies (9 prospective cohort studies [9, 13, 15, 17, 22–26] and 17 retrospective cohort studies [10–12, 14, 16, 27–38]) for the meta-analysis. Among these 26 studies, 20 compared allo-HSCT group to CMT group [9–12, 15–17, 23, 24, 26–34, 36, 37], 9 compared allo-HSCT group to auto-HSCT group [9, 13–15, 17, 22, 25, 35, 38] and 3 compared auto-HSCT group to CMT group [9, 15, 17]. Quality assessment of included studies are generally determined to be good except some studies which were conference abstracts [25, 28, 35]. The literature review and selection process are described in Fig 1.

### Baseline patient characteristics

All 26 eligible studies consisted of 1,522 allo-HSCT recipients, 215 auto-HSCT recipients, and 838 patients who did not receive any HSCT. Age of participants covered all age groups with a median of approximately 30–60 years old, generally with older patients in CMT group than those in allo-HSCT and auto-HSCT groups [10, 14–16, 22, 27, 29, 31, 34, 36, 37]. Out of 26 studies, 23 studies had the entire cohort receiving TKIs during the study period [9–13, 15–17, 22–26, 28, 29, 31–38] and 12 studies included only patients who achieved CR1 before HSCT or CMT for analysis [10, 11, 14, 16, 24–26, 30, 33, 34, 36, 37]. 12 studies had only newly-diagnosed Ph^+^ ALL patients [9, 11, 13–15, 17, 24, 26, 30, 33, 34, 36] and one study included CML patients with Ph^+^ ALL blast crisis [28]. Basic characteristics and quality assessment results of included studies are summarized in Tables 1–3. The individual components of the study quality assessment are presented in S1 Table.

**Table 1:** Characteristics and participants of studies that compare allogeneic HSCT to chemotherapy.

References	Group	No.	Sex (M/ F)	Median age (years, range)	TKI (type and dosage)	Chemotherapy regimen before HSCT	Conditioning regimen (MAC/RIC/non-MAC)	Donor type (MSD/MUD/Haplo/CBT)	Study period/Median F/U	Type	Quality assessment
Bassan 2010 [17]	Allo	34	NA	NA	Imatinib 600 mg/day for the first 7 days then after 3 days before CMT	NILG protocol 09/00	34/0/0	17/17/0/0	2000–2010	Prospective cohort study	S: 4
	CMT	15	NA	NA	Imatinib 600 mg/day for the first 7 days then after 3 days before CMT	NILG protocol 09/00	-	-			C: 2
											O: 3
Li 2010 [27]	Allo	22	16/6	32 (16–51)	Imatinib 600 mg/day starting 24 hours after completing CMT until the beginning of the next course	Five-drug (vincristine, daunorubicin, cyclophosphamide, prednisone and L-asparagine) induction therapy, then consolidation and maintenance therapy	NA	18/4/0/0	June 1996 to December 2007	Retrospective cohort study	S: 3
	CMT	41	29/12	36 (15–59)	Imatinib 600 mg/day starting 24 hours after completing CMT until the beginning of the next course	Five-drug (vincristine, daunorubicin, cyclophosphamide, prednisone and L-asparagine) induction therapy, then consolidation and maintenance therapy	-	-			C: 0
											O: 3
Pfeifer 2012 [28]	Allo	11	NA	NA	Imatinib	NA	NA	NA	NA	Retrospective cohort study	S: 0
	CMT	104	NA	NA	Imatinib	NA	-	-			C: 1
											O: 1
Konopacki 2013 [29]	Allo	10	NA	46	Imatinib or dasatinib	Hyper-CVAD or GRAALL protocol	NA	5/3/0/2	March 2004 –July 2012	Retrospective cohort study	S: 3
	CMT	8	NA	63	Imatinib or dasatinib	Hyper-CVAD or GRAALL protocol	-	-			C: 1
											O: 3
Tanguy-Schmidt 2013 [15]	Allo	24	14/10	40 (16–56)	Imatinib 600–800 mg/day	GRAAPH-2003 induction and consolidation therapy	24/0/0	15/9/0/0	January 2004 to October 2005	Prospective cohort study	S: 4
	CMT	9	5/4	50 (42–55)	Imatinib 600–800 mg/day	GRAAPH-2003 induction and consolidation therapy	-	-			C: 2
											O: 3
Fielding 2014 [9]	Allo	87	NA	NA	Imatinib 400–600 mg/day	NA	76/11/0	43/33/0/0	March 2003 –October 2008	Prospective cohort study	S: 4
	CMT	38	NA	NA	Imatinib 400–600 mg/day	NA	-	-			C: 2
											O: 3
Daver 2015 [23]	Allo	16	NA	NA	Imatinib 400–600 mg/day on day 1–14 of each cycle and 600–800 mg/day during maintenance therapy	Hyper-CVAD	NA	10/5/0/1	April 2001 –November 2006	Prospective cohort study	S: 3
	CMT	23	NA	NA	Imatinib 400–600 mg/day on days 1–14 of each cycle and 600–800 mg/day during maintenance therapy	Hyper-CVAD	-	-			C: 1
											O: 3
Ravandi 2015 [24]	Allo	12	NA	NA	Dasatinib 100 mg/day daily on days 1–14 of the first cycle followed by 70 mg/day from the second cycle	Hyper-CVAD	12/0/0	7/4/0/1	September 2006 –March 2012	Prospective cohort study	S: 4
	CMT	60	NA	NA	Dasatinib 100 mg/day daily on days 1–14 of the first cycle followed by 70 mg/day from the second cycle	Hyper-CVAD	-	-			C: 2
											O: 3
Sun 2015 [30]	Allo	30	NA	NA	Imatinib 400 mg/day	Four-drug (vincristine, daunorubicin, cyclophosphamide and prednisone) induction therapy, then consolidation and maintenance therapy	30/0/0	0/0/30/0	January 2000 –December 2012	Retrospective cohort study	S: 4
	CMT	32	NA	NA	Imatinib 400 mg/day	Four-drug (vincristine, daunorubicin, cyclophosphamide and prednisone) induction therapy, then consolidation and maintenance therapy	-	-			C: 1
											O: 3
Togasaki 2015 [31]	Allo	13	9/4	39 (22–60)	Imatinib 600 mg/day	Hyper-CVAD or Ph-positive ALL 202 protocol	11/2/0	2/8/0/3	March 2002 –June 2011	Retrospective cohort study	S: 3
	CMT	9	3/6	60 (55–72)	Imatinib 600 mg/day	Hyper-CVAD or Ph-positive ALL 202 protocol	-	-			C: 1
											O: 3
Kanfar 2016 [32]	Allo	59	NA	NA	Imatinib	DFCI pediatric ALL protocol	NA	NA	2001–2015	Retrospective cohort study	S: 3
	CMT	74	NA	NA	Imatinib	DFCI pediatric ALL protocol	-	-			C: 1
											O: 3
Kuang 2016 [33]	Allo	6	NA	NA	Imatinib 400 mg/day	Two-drug (vincristine, dexamethasone) induction therapy, then consolidation and maintenance therapy	NA	NA	October 2008 –June 2012	Retrospective cohort study	S: 4
	CMT	43	NA	NA	Imatinib 400 mg/day	Two-drug (vincristine, dexamethasone) induction therapy, then consolidation and maintenance therapy	-	-			C: 2
											O: 3
Fujisawa 2017 [34]	Allo	43	25/18	41 (18–61)	Imatinib 600 mg/day	Four-drug (daunorubicin, cyclophosphamide, vincristine, and prednisolone) induction therapy, then consolidation and maintenance therapy	NA	13/23/0/7	October 2008—December 2010	Retrospective cohort study	S: 4
	CMT	22	7/15	61 (19–64)	Imatinib 600 mg/day	Four-drug (daunorubicin, cyclophosphamide, vincristine, and prednisolone) induction therapy, then consolidation and maintenance therapy	-	-			C: 2
											O: 3
Kozlowski 2017 [10]	Allo	14	7/7	59.5 (55–65)	Imatinib or dasatinib	EWALL-backbone therapy, ABCDV protocol, hyper-CVAD, or daunorubicin/cytara-bine induction therapy	NA	NA	2005–2012	Retrospective cohort study	S: 3
	CMT	28	12/16	67.5 (58–82)	Imatinib or dasatinib	EWALL-backbone therapy, ABCDV protocol, hyper-CVAD, or daunorubicin/cytara-bine induction therapy	-	-			C: 2
											O: 2
Hatta 2018 [11]	Allo	59	NA	NA	Imatinib	Ph-positive ALL 202 protocol	NA	30/21/0/8	August 2002 –May 2005	Retrospective cohort study	S: 4
	CMT	37	NA	NA	Imatinib	Ph-positive ALL 202 protocol	-	-			C: 2
											O: 3
Jabbour 2018 [26]	Allo	15	NA	NA	Ponatinib 45 mg/day on D1-14 of 1^st^ cycle then 30 mg/day continuously from cycle 2, and 15 mg/day if achieve CMR	Hyper-CVAD	NA	8/5/2/0	November 2011—April 2018	Prospective cohort study	S: 4
	CMT	47	NA	NA	Ponatinib 45 mg/day on D1-14 of 1^st^ cycle then 30 mg/day continuously from cycle 2, and 15 mg/day if achieve CMR	Hyper-CVAD	-	-			C: 2
											O: 3
Agrawal 2019 [12]	Allo	16	NA	NA	Imatinib 400 mg/day or dasatinib 140 mg/day	COG0232 or UK-ALL protocols	9/4/3	12/0/4/0	January 2011 –June 2016	Retrospective cohort study	S: 3
	CMT	35	NA	NA	Imatinib 400 mg/day or dasatinib 140 mg/day	COG0232 or UK-ALL protocols	-	-			C: 1
											O: 3
Chang 2019 [16]	Allo	30	21/9	42 (21–65)	Dasatinib 140 mg/day	Hyper-CVAD, BFM‐like, or pediatric‐inspired ALL regimen	30/0/0	14/7/9/0	2005–2018	Retrospective cohort study	S: 3
	CMT	40	24/16	44 (21–69)	Dasatinib 140 mg/day	Hyper-CVAD, BFM‐like, or pediatric‐inspired ALL regimen	-	-			C: 2
											O: 3
Ghobadi 2020 [36]	Allo	66	45/21	45 (19–71)	Imatinib, dasatinib, or ponatinib	Hyper-CVAD	57/9/0	NA	January 2001—December 2018	Retrospective cohort study	S: 4
	CMT	120	57/63	56 (19–84)	Imatinib, dasatinib, or ponatinib	Hyper-CVAD	-	-			C: 2
											O: 3
Wang 2020 [37]	Allo	60	39/21	36 (15–59)	Imatinib 400–600 mg/day or dasatinib 100 mg/day	Hyper-CVAD, or three-drug (daunorubicin, vincristine, and prednisolone) induction therapy	60/0/0	NA	January 2007—December 2017	Retrospective cohort study	S: 3
	CMT	74	32/42	40.5 (14–60)	Imatinib 400–600 mg/day or dasatinib 100 mg/day	Hyper-CVAD, or three-drug (daunorubicin, vincristine, and prednisolone) induction therapy	-	-			C: 2
											O: 3

**Abbreviations:** *ABCDV* cytarabine, betamethasone, cyclophosphamide, daunorubicin and vincristine, *ALL* acute lymphoblastic leukemia, *Allo* allogeneic hematopoietic stem cell transplantation, *C* compatibility, *BFM* Berlin-Frankfurt-Münster, *CBT* cord blood transplantation, *CMR* complete molecular response, *CMT* chemotherapy, *COG0232* Children’s Oncology Group AALL0232, *DFCI* Dana Farber Cancer Institute, *EWALL* European Working Group on Adult ALL, *F* female, *GRAAPH* Group for Research on Adult Acute Lymphoblastic Leukemia Philadelphia positive, *Haplo* haploidentical, *HSCT* hematopoietic stem cell transplantation, *Hyper-CVAD* hyperfractionated cyclophosphamide, vincristine, doxorubicin and dexamethasone, *MAC* myeloablative conditioning, *M* male, *MSD* matched sibling donor, *MUD* match unrelated donor, *NA* not available, *NILG* Northern Italy Leukemia Group, *No*. number of participants, *O* outcome, *RIC* reduced intensity conditioning, *S* selection, *TKI* tyrosine kinase inhibitor, *UK-ALL* United Kingdom Acute Lymphoblastic Leukemia

**Table 2:** Characteristics and participants of studies that compare allogeneic HSCT to autologous HSCT.

References	Group	No.	Sex (M/ F)	Median age (years, range)	TKI (type and dosage)	Chemotherapy regimen before HSCT	Conditioning regimen (MAC/RIC/non-MAC)	Donor type (MSD/MUD/Haplo/CBT)	Study period/Median F/U	Type	Quality assessment
Bassan 2010 [17]	Allo	34	NA	NA	Imatinib 600 mg/day for the first 7 days then after 3 days before CMT	NILG protocol 09/00	34/0/0	17/17/0/0	2000–2010	Prospective cohort study	S: 4
	Auto	5	NA	NA	Imatinib 600 mg/day for the first 7 days then after 3 days before CMT	NILG protocol 09/00	5/0/0	-			C: 2
											O: 3
Tanguy-Schmidt 2013 [15]	Allo	24	14/10	40 (16–56)	Imatinib 600–800 mg/day	GRAAPH-2003 induction and consolidation therapy	24/0/0	15/9/0/0	January 2004—October 2005	Prospective cohort study	S: 4
	Auto	10	4/6	44 (27–59)	Imatinib 600–800 mg/day	GRAAPH-2003 induction and consolidation therapy	10/0/0	-			C: 2
											O: 3
Fielding 2014 [9]	Allo	87	NA	NA	Imatinib 400–600 mg/day	NA	76/11/0	43/33/0/0	March 2003 –October 2008	Prospective cohort study	S: 4
	Auto	5	NA	NA	Imatinib 400–600 mg/day	NA	76/11/0	-			C: 2
											O: 3
Wetzler 2014 [22]	Allo	15	7/8	43 (26–54)	Imatinib 800 mg/day before HSCT and 400 mg/day after HSCT for at least 12 months until two negative RT-PCR or until relapse	Protocol course I-VI	15/0/0	15/0/0/0	April 2002—April 2010	Prospective cohort study	S: 3
	Auto	19	9/10	49 (24–57)	Imatinib 800 mg/day before HSCT and 400 mg/day after HSCT for at least 12 months until two negative RT-PCR or until relapse	Protocol course I-VI	19/0/0	-			C: 1
											O: 3
Chalandon 2015 [13]	Allo	161	NA	NA	Imatinib 600–800 mg/day	GRAAPH-2005 treatments	124/37/0	76/72/0/13	May 2006- August 2011	Prospective cohort study	S: 4
	Auto	35	NA	NA	Imatinib 600–800 mg/day	GRAAPH-2005 treatments	35/0/0	-			C: 2
											O: 3
Tan 2015 [25]	Allo	34	NA	NA	Imatinib post-transplant	NA	32/2/0	30/2/2/0	January 2007- December 2014	Prospective cohort study	S: 0
	Auto	2	NA	NA	Imatinib post-transplant	NA	2/0/0	-			C: 2
											O: 1
Liu 2017 [35]	Allo	55	NA	NA	Imatinib, nilotinib, or dasatinib	NA	NA	55/0/0/0	May 2005- December 2016	Retrospective cohort study	S: 0
	Auto	31	NA	NA	Imatinib, nilotinib, or dasatinib	NA	NA	-			C: 1
											O: 1
Giebel 2018 [14]	Allo	502	262/238	40 (18–65)	NA	NA	502/0/0	255/247/0/0	2007–2014	Retrospective cohort study	S: 4
	Auto	67	37/30	46 (20–65)	NA	NA	6/0/0	-			C: 1
											O: 3
Lyu 2021 [38]	Allo	77	45/32	NA	Imatinib 400–600 mg/day, dasatinib 100–120 mg/day, nilotinib 600–800 mg/day	VDCP regimen	77/0/0	60/0/17/0	January 2008- October 2019	Retrospective cohort study	S: 3
	Auto	42	30/12	NA	Imatinib 400–600 mg/day, dasatinib 100–120 mg/day, nilotinib 600–800 mg/day	VDCP regimen	42/0/0	-			C: 1
											O: 3

**Abbreviations:** *Allo* allogeneic hematopoietic stem cell transplantation, *Auto* autologous hematopoietic stem cell transplantation, *C* compatibility, *CBT* cord blood transplantation, *CMT* chemotherapy, *F* female, *GRAAPH* Group for Research on Adult Acute Lymphoblastic Leukemia Philadelphia positive, *Haplo* haploidentical, *HSCT* hematopoietic stem cell transplantation, *MAC* myeloablative conditioning, *M* male, *MSD* matched sibling donor, *MUD* match unrelated donor, *NA* not available, *NILG* Northern Italy Leukemia Group, *No*. number of participants, *O* outcome, *RIC* reduced intensity conditioning, *S* selection, *TKI* tyrosine kinase inhibitor, *VDCP* vincristine, daunorubicin, cyclophosphamide, and prednisone.

**Table 3:** Characteristics and participants of studies that compare autologous HSCT to chemotherapy alone.

References	Group	No.	Sex (M/ F)	Median age (years, range)	TKI (type and dosage)	Chemotherapy regimen before HSCT	Conditioning regimen (MAC/RIC/non-MAC)	Study period	Type	Quality assessment
Bassan 2010 [17]	Auto	5	NA	NA	Imatinib 600 mg/day for the first 7 days then after 3 days before CMT	NILG protocol	5/0/0	2000–2010	Prospective cohort study	S: 4
	CMT	15	NA	NA	Imatinib 600 mg/day for the first 7 days then after 3 days before CMT	NILG protocol	-			C: 2
										O: 3
Tanguy-Schmidt 2013 [15]	Auto	10	4/6	44 (27–59)	Imatinib 600–800 mg/day	GRAAPH-2003 induction and consolidation therapy	10/0/0	January 2004 to October 2005	Prospective cohort study	S: 4
	CMT	9	5/4	50 (42–55)	Imatinib 600–800 mg/day	GRAAPH-2003 induction and consolidation therapy	-			C: 2
										O: 3
Fielding 2014 [9]	Auto	5	NA	NA	Imatinib 400–600 mg/day	NA	76/11/0	March 2003 –October 2008	Prospective cohort study	S: 4
	CMT	38	NA	NA	Imatinib 400–600 mg/day	NA	-			C: 2
										O: 3

**Abbreviations:** *Auto* autologous hematopoietic stem cell transplantation, *CMT* chemotherapy, *F* female, *GRAAPH* Group for Research on Adult Acute Lymphoblastic Leukemia Philadelphia positive, *Haplo* haploidentical, *HSCT* hematopoietic stem cell transplantation, *inter* intermediate, *MAC* myeloablative conditioning, *M* male, *MSD* matched sibling donor, *MUD* match unrelated donor, *NA* not available, *NILG* Northern Italy Leukemia Group, *No*. number of participants, *O* outcome, *RIC* reduced intensity conditioning, *S* selection, *TKI* tyrosine kinase inhibitor

### Drug regimens used during induction and maintenance

A wide range of chemotherapeutic regimens were employed; the most common regimens are hyper-CVAD (hyperfractionated cyclophosphamide, vincristine, doxorubicin, dexamethasone), multiagent CMT (vincristine, daunorubicin, prednisolone-based), pediatric-inspired and GRAAPH regimens. For TKI treatment, imatinib was the most commonly used in 22 studies, with a dose range of 400–800 mg/day [9–13, 15, 17, 22, 23, 25, 27–38]. Dasatinib is used in nine studies, with a dose range of 70–140 mg/day [10, 12, 16, 24, 29, 35–38], ponatinib is used in two studies, with a dose range of 15–45 mg/day [26, 36], and nilotinib is used in two studies, with a dose range of 600–800 mg/day [35, 38]. Regarding post-transplant TKI, it is given to all patients in nine studies [9, 12, 16, 25, 27, 30, 33, 37], to some patients in six studies [13–15, 26, 36, 38], to none of the patients in one study [31], and no report of post-transplant TKI in ten studies [10, 11, 17, 23, 24, 28, 29, 32, 34, 35].

### Characteristics of HSCT procedures

Among all allo-HSCT patients, 685 had matched-sibling donors, 460 had matched-unrelated donors, 64 had haploidentical donors and 35 had umbilical cord blood transplantation. For conditioning regimens, 1,093 allo- HSCT and 185 auto-HSCT patients received myeloablative conditioning, while 65 allo-HSCT and 11 auto-HSCT patients received reduced-intensity conditioning. For both allo-HSCT and auto-HSCT, the majority of patients had total body irradiation (TBI)-based regimens (such as TBI/cyclophosphamide, TBI/fludarabine, and TBI/etoposide), followed by busulfan-based regimens (such as busulfan/cyclophosphamide, busulfan/fludarabine, and busulfan/melphalan).

### Outcomes of allo-HSCT versus CMT on Ph^+^ ALL patients

For the outcomes of allo-HSCT and CMT groups, a total of 19 and 13 studies reported OS rates (as 2-year rate by one study [27], 2.5-year rate by one study [29], 3-year rate by six studies [10, 16, 26, 30, 33, 34], 4-year rate by four studies [9, 12, 15, 37] and 5-year rate by seven studies [11, 17, 24, 28, 31, 32, 36]) and DFS rates (as 2-year rate by one study [26], 3-year rate by three studies [16, 30, 33], 4-year rate by four studies [9, 12, 15, 37] and 5-year rate by five studies [11, 17, 23, 32, 36]), respectively. Two and nine studies reported TRM rates (as 4-year rate by one study [15] and 5-year rate by one study [31]) and CIR rates (8-month rate by one study [29], 2-year rate by one study [27], 3-year rate by one study [30], 4-year rate by three studies [12, 15, 37] and 5-year rate by three studies [11, 31, 36]), respectively.

**Fig 2:**
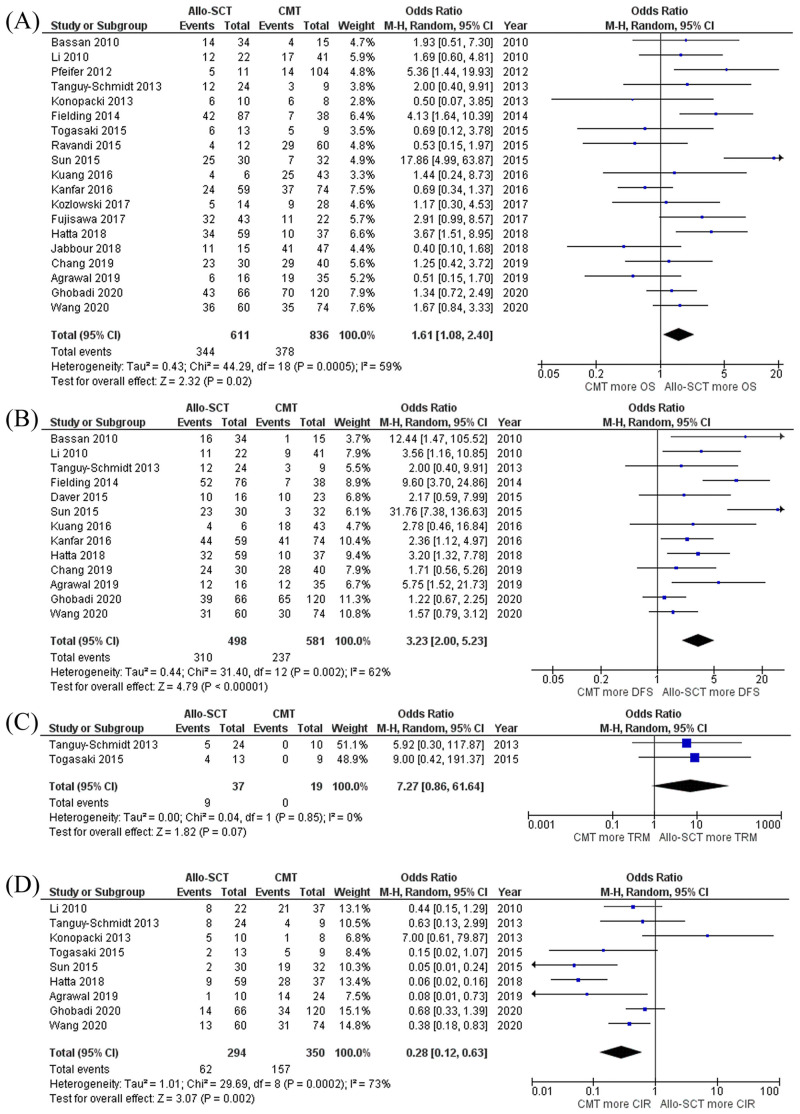
Forest plot of the meta-analysis of allo-HSCT versus CMT only. (A) OS rate (B) DFS rate (C) TRM rate (D) CIR rate. **Abbreviations**: *Allo-SCT* Allogenic stem cell transplantation, *CIR* Cumulative incidence of relapse, *CMT* Chemotherapy alone, *DFS* Disease-free survival, *OS* Overall survival, *TRM* Treatment-realated mortality.

The pooled meta-analysis showed that Ph^+^ ALL patients who received allo-HSCT had a significantly prolonged OS and DFS compared to those who received only CMT with pooled odds ratio (OR) of 1.61 (95% CI, 1.08–2.40; I^2^ = 59%) and 3.23 (95% CI, 2.00–5.23; I^2^ = 62%), respectively (Fig 2A and 2B). However, patients who received allo-HSCT also had a higher incidence of TRM but lower CIR than patients who did not receive any HSCT with pooled OR of 7.27 (95% CI, 0.86–61.64; I^2^ = 0%) and 0.28 (95% CI, 0.12–0.63; I^2^ = 73%), respectively (Fig 2C and 2D). The causes of TRM were not mentioned in the included studies. Funnel plots of OS and DFS were relatively symmetric and showed no presence of publication bias. **(Fig 5A, 5B)**.

### Outcomes of allo-HSCT versus auto-HSCT on Ph^+^ ALL patients

For the outcomes of allo-HSCT and auto-HSCT groups, a total of nine and eight studies reported OS rates (as 2-year rate by one study [14], 3-year rate by three studies [25, 35, 38], 4-year rate by two studies [9, 15] and 5-year rate by three studies [13, 17, 22]) and DFS (as 2-year rate by one study [14], 3-year rate by two studies [35, 38], 4-year rate by two studies [9, 15] and 5-year rate by three studies [13, 17, 22]), respectively. Four and six studies reported TRM rates (specified as 100-day rate by two studies [22, 25], 4-year rate by one study [15] and one study without specified duration [35]) and CIR rates (2-year rate by one study [14], 3-year rate by one study [38], 4-year rate by one study [15], 5-year rate by two studies [13, 22] and one study without specified duration [35]), respectively.

**Fig 3:**
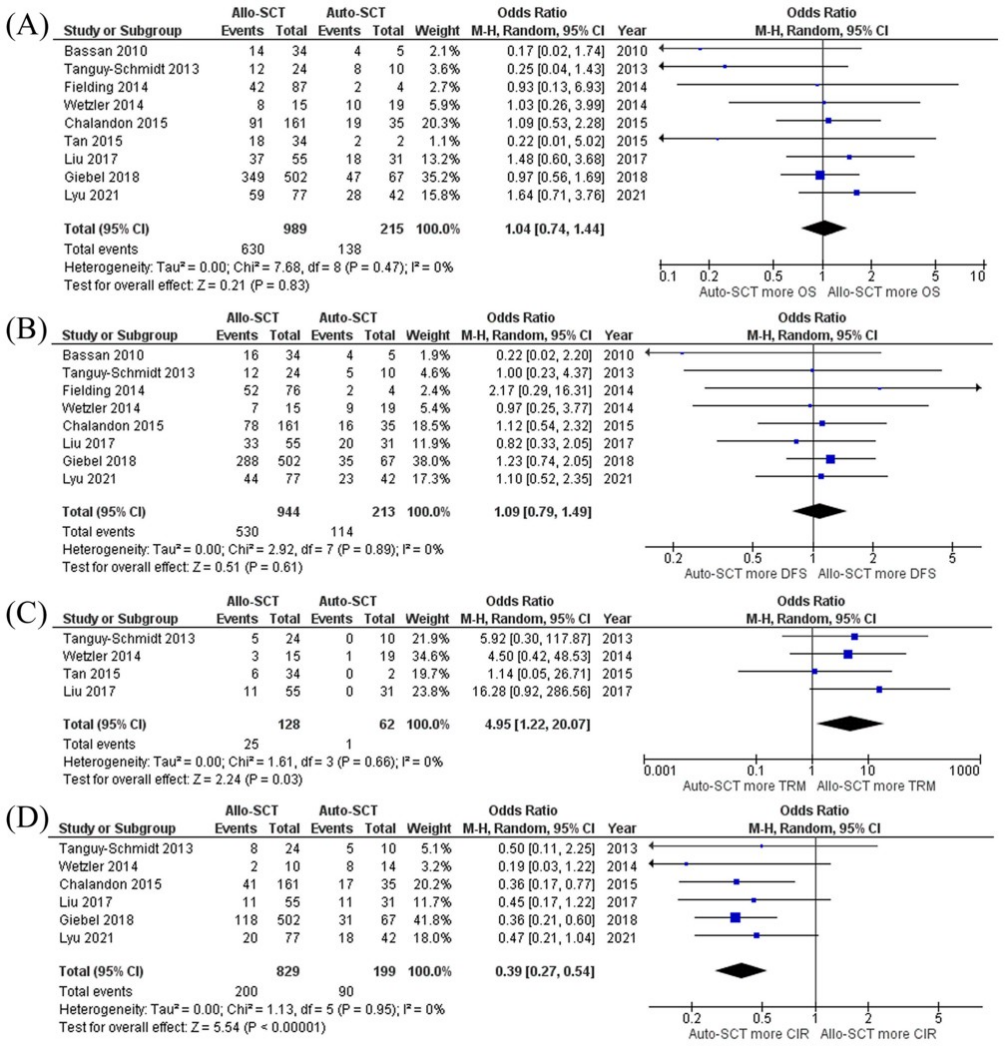
Forest plot of the meta-analysis of allo-HSCT versus auto-HSCT. (A) OS rate (B) DFS rate (C) TRM rate (D) CIR rate. **Abbreviations**: *Allo-SCT* Allogenic stem cell transplantation, *Auto-SCT* Autologous stem cell transplantation, *CIR* Cumulative incidence of relapse, *DFS* Disease-free survival, *OS* Overall survival, *TRM* Treatment-realated mortality.

The pooled meta-analysis found that Ph^+^ ALL patients who received allo-HSCT had comparable OS and DFS to those who received auto-HSCT with pooled OR of 1.04 (95% CI, 0.74–1.44; I^2^ = 0%) and 1.09 (95% CI, 0.79–1.49; I^2^ = 0%), respectively (Fig 3A and 3B). However, patients who received allo-HSCT had an increased TRM rate but decreased CIR rate than patients who received auto-HSCT with pooled OR of 4.95 (95% CI, 1.22–20.07; I^2^ = 0%) and 0.39 (95% CI, 0.27–0.54; I^2^ = 0%), respectively (Fig 3C and 3D). The causes of TRM were mainly veno-occlusive disease, opportunistic infection, and graft-versus-host disease. The funnel plot of OS was asymmetric, which suggested the possibility of publication bias that favors auto-HSCT to allo-HSCT. On the other hand, the funnel plot of DFS was fairly symmetric and not suggestive of the presence of publication bias. **(Fig 5C, 5D)**.

A subgroup analysis by the number of years of OS and DFS was performed to exclude the possibility of similar survival outcomes due to the included studies having too short follow-up time. There were no statistical difference between each subgroup (p = 0.39 for OS and p = 0.90 for DFS) (S1A and S1B Fig).

### Outcomes of auto-HSCT versus CMT on Ph^+^ ALL patients

**Fig 4:**
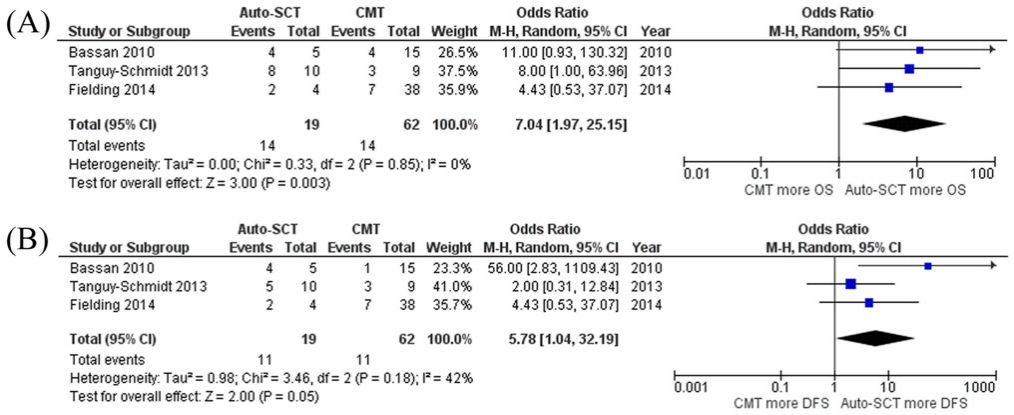
Forest plot of the meta-analysis of auto-HSCT versus CMT only. (A) OS rate (B) DFS rate. **Abbreviations**: *Auto-SCT* Autologous stem cell transplantation, *CMT* Chemotherapy only, *DFS* Disease-free survival, *OS* Overall surviva.

**Fig 5:**
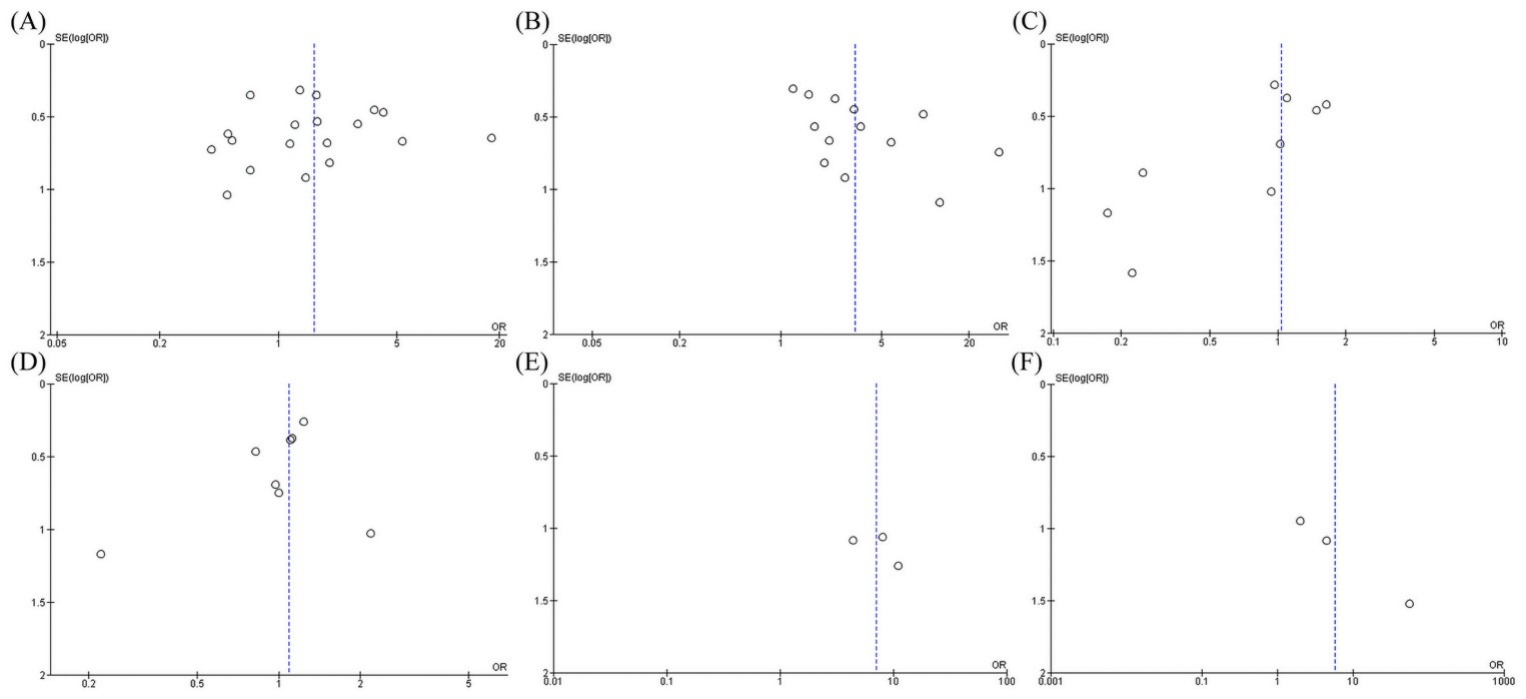
Funnel plot of the meta-analysis of (A) OS rate between allo-HSCT versus CMT only, (B) DFS rate between allo-HSCT versus CMT (C) OS rate between allo-HSCT versus auto-HSCT (D) DFS rate between allo-HSCT versus auto-HSCT (E) OS rate between auto-HSCT versus CMT (F) DFS rate between auto-HSCT versus CMT only.

For the outcomes of auto-HSCT and CMT groups, a total of three studies reported OR and DFS rates (as 4-year rate by two studies [9, 15], and 5-year rate by one study [17]). The pooled meta-analysis showed that Ph^+^ ALL patients who received auto-HSCT had significantly sustained OS and DFS compared to those who received only CMT with pooled odds ratio (OR) of 7.04 (95% CI, 1.97–25.15; I^2^ = 0%) and 5.78 (95% CI, 1.04–32.19; I^2^ = 42%), respectively (Fig 4A and 4B). Funnel plots of OS and DFS were fairly symmetrical, which was not suggestive of the presence of publication bias (Fig 5E and 5F).

### Subgroup analysis of studies with all Ph^+^ ALL patients receiving TKIs

Subgroup analysis was conducted based on studies with all Ph^+^ ALL patients receiving TKI. Pooled OR of OS and DFS between allo-HSCT group and CMT group were slightly decreased to 1.42 (95% CI, 0.98–2.05; I^2^ = 46%) [9–12, 15–17, 24, 26, 28, 29, 31–34, 36, 37] and 2.64 (95% CI, 1.71–4.07; I^2^ = 48%) [9, 11, 12, 15–17, 23, 32, 33, 36, 37], respectively, but still attained statistical significance (S2A and S2B Fig). However, pooled results between allo-HSCT group and auto-HSCT group were not significantly changed (pooled OR of OS 1.05; 95% CI, 0.67–1.63; I^2^ = 8% [9, 13, 15, 17, 22, 25, 35, 38] and pooled OR of DFS 1.00; 95% CI, 0.67–1.50; I^2^ = 0% [9, 13, 15, 17, 22, 35, 38]) (S2C and S2D Fig).

### Subgroup analysis of studies with all Ph^+^ ALL patients receiving post-transplant TKIs

Subgroup analysis of studies with all Ph+ ALL patients receiving post-transplant TKIs in the allo-HSCT group versus CMT group showed increased pooled OR of OS and DFS to 2.12 (95% CI, 1.01–4.46; I^2^ = 70%) and 4.39 (95% CI, 1.99–9.69; I^2^ = 71%), respectively [9, 12, 16, 27, 30, 33, 37] (S3A and S3B Fig). On the other hand, the pooled OS and DFS in the allo-HSCT group versus auto-HSCT group were not different (pooled OR of OS and DFS 0.84; 95% CI, 0.29–2.41; I^2^ = 0% [9, 22, 25] and 1.25; 95% CI, 0.40–3.85; I^2^ = 0% [9, 22], respectively) (S3C and S3D Fig).

### Subgroup analysis of studies with all Ph^+^ ALL patients achieving CR1 before HSCT/CMT

Subgroup analysis of studies with all Ph^+^ ALL patients achieving CR1 prior to allo-HSCT or CMT showed increased pooled OR of OS to 1.75 (95% CI, 1.00–3.06; I^2^ = 64%) [10, 11, 16, 24, 26, 30, 33, 34, 36, 37] while pooled OR of DFS was decreased to 2.69 (95% CI, 1.27–5.72; I^2^ = 73%) [11, 16, 26, 30, 33, 36, 37] (S4A and S4B Fig). In addition, the pooled result between allo-HSCT group and auto-HSCT group were not significantly different (pooled OR of OS 0.93; 95% CI, 0.54–1.60; I^2^ = 0%) [14, 25] (S4C Fig).

### Subgroup analysis of studies by type of TKI

Subgroup analysis of studies by type of TKI used in each study showed that later generations of TKI, namely, dasatinib and ponatinib, pooled OR of OS in CMT group was comparable to allo-HSCT group (pooled OR of OS for dasatinib 0.88; 95% CI, 0.38–2.03; I^2^ = 0% [16, 24] and for ponatinib 0.40; 95% CI, 0.10–1.68; I^2^ not applicable [26]). Similarly, pooled OR of DFS was not different between both groups for dasatinib (pooled OR 1.71; 95% CI, 0.56–5.26; I^2^ not applicable) [16]. On the other hand, the benefit of allo-HSCT compared to CMT was considerably increased in patients receiving imatinib (pooled OR of OS and DFS for imatinib 2.48; 95% CI, 1.38–4.48; I^2^ = 65% [9, 11, 15, 17, 27, 28, 30–34] and 4.40; 95% CI, 2.48–7.80; I^2^ = 50% [9, 11, 15, 17, 23, 27, 30, 32, 33], respectively) (S5A and S5B Fig).

## Discussion

Our systematic review and meta-analysis showed that HSCT remains the most favorable therapeutic strategies as it reveals superior OS, DFS and relapse rate compared to CMT alone. This confirms the benefit and necessity of HSCT in combination with TKIs for the treatment of adult Ph^+^ ALL patients despite the remarkable efficacy of TKI in these patients.

Although allogeneic HSCT, especially haploidentical HSCT, can be useful for finding potential stem cell donors, its costliness and potential complications during HSCT could hinder the usage of the procedure for various countries. Therefore, auto-HSCT could be a reasonable choice for adult Ph^+^ ALL patients who are ineligible for allo-HSCT or have no donor since it could provide comparable survival outcomes to allo-HSCT, although higher incidence of relapse is found in auto-HSCT group. With unmet needs for hematopoietic stem cell donors, auto-HSCT might be beneficial for patients in this type of situation [39, 40].

Interestingly, several studies have shown that patients who attained the first deeper molecular response, such as major and complete molecular response, had similar outcomes regardless of the status of transplantation. Results also suggested that negative minimal residual disease (MRD) status is also predictive of better survival outcomes and lower incidence of relapse compared to those with positive MRD in Ph^+^ ALL, suggesting the potential of utilizing MRD status to classify patient subpopulation who would benefit from allo-HSCT [41–43]. Unfortunately, the data are still limited and more studies should explore further this subgroup of patients [11, 14, 16, 34, 37].

Moreover, our study results supported the stronger efficacy of dasatinib and ponatinib than imatinib, in line with the result from a recent study [43]. However, the data on comparing the efficacy between each TKI are scarce, and more head-to-head studies are needed to investigate this issue.

There have been interests in combining chemotherapy with immunotherapy such as blinatumomab and inotuzumab ozogamicin, and later generations of TKIs with greater potency such as ponatinib and asciminib to overcome resistance mutations such as T315I [44–46]. This could help patients achieving negative minimal residual disease status, leading to better outcomes and even dismissing the need for HSCT [43]. A phase 3 randomized controlled study comparing ponatinib and imatinib induction, consolidation, and maintenance in Ph^+^ ALL patients is ongoing (NCT03589326) [47]. Moreover, recent advancements in chemotherapy-free induction and consolidation regimens also showed promising results. A single-armed phase 2 study conducted by GIMEMA group, using dasatinib and blinatumomab, showed favorable outcomes in both the high rate of molecular response and survival outcomes [48]. Phase 3 trials comparing between blinatumomab and chemotherapy in Ph^+^ ALL patients who receive steroids and TKIs are currently underway (NCT04530565, NCT04722848) [49, 50].

Some limitations could hinder the interpretation of the results. Firstly, the heterogeneity found in studies that were included in this meta-analysis is due to several factors including population characteristics, differences in study design, chemotherapeutic and TKI regimens, and transplantation procedures. Some studies included non-CR1 patients and relapsed/refractory Ph^+^ ALL, which were associated with poorer survival outcomes [42, 51]. Moreover, some studies included a minority of patients who did not receive any TKIs in the analysis which could affect the result in favor of HSCT [14, 27, 30]. Various ablative regimens used in the included studies and the use of post-transplant TKI could also play role in survival outcomes and confounded the result.

Secondly, there could be selection bias as older patients, which are associated with poorer outcomes, would tend to be unfit for transplantation, leading them to receive CMT alone instead of transplantation [9]. This was observed in multiple studies included in this meta-analysis. However, the small number of studies that reported the age of participants in each arm limit the ability to perform a sensitivity analysis in this systematic review and meta-analysis.

Third, most of the included studies did not include analyses based on the MRD status. As positive MRD status is associated with poorer survival outcomes and greater incidence of relapse, this could affect the benefit of HSCT and confound the result of the meta-analysis [41–43].

Finally, a small number of studies could underpower the results of the subgroup analyses and cause non-significance. Along with the fact that all of the studies are cohort studies, more randomized controlled trials are certainly needed to confirm the efficacy of HSCT in Ph^+^ ALL patients.

## Conclusion

This systematic review and meta-analysis exhibit superior results of HSCT with TKIs in adult Ph^+^ ALL patients compared to CMT with TKIs and endorse the utilization of HSCT in this group of patients who are fit for transplantation. Auto-HSCT could be performed instead of allo-HSCT with comparable survival outcomes in patients who had no available donor and haploidentical HSCT was not feasible. However, more randomized controlled studies are still required to confirm the comparable efficacy of auto-HSCT and allo-HSCT; the role of MRD-guided treatment strategies on the efficacy of transplantation versus CMT should also be investigated.

## Supporting information

S1 File Searching strategy. (DOCX)

S1 Checklist PRISMA checklist. (DOCX)

S1 Table Individual component of the study quality assessment. (DOCX)

S1 Fig Subgroup analysis of studies by the number of years of survival rate. (A) OS rate between allo-HSCT versus auto-HSCT (B) DFS rate between allo-HSCT versus auto-HSCT.
(TIF)

S2 Fig Subgroup analysis of studies with all Ph^+^ ALL patients receiving TKIs. (A) OS rate between allo-HSCT versus CMT only (B) DFS rate between allo-HSCT versus CMT (C) OS rate between allo-HSCT versus auto-HSCT (D) DFS rate between allo-HSCT versus auto-HSCT.
(TIF)

S3 Fig Subgroup analysis of studies with all Ph^+^ ALL patients receiving post-transplant TKIs. (A) OS rate between allo-HSCT versus CMT only (B) DFS rate between allo-HSCT versus CMT (C) OS rate between allo-HSCT versus auto-HSCT (D) DFS rate between allo-HSCT versus auto-HSCT.
(TIF)

S4 Fig Subgroup analysis of studies with all Ph^+^ ALL patients achieving CR1 before HSCT/CMT. (A) OS rate between allo-HSCT versus CMT only (B) DFS rate between allo-HSCT versus CMT (C) OS rate between allo-HSCT versus auto-HSCT.
(TIF)

S5 Fig Subgroup analysis of studies by type of TKI. (A) OS rate between allo-HSCT versus CMT only (B) DFS rate between allo-HSCT versus CMT.
(TIF)
