# Supplementary material for: Is stem cell transplantation still needed for adult Philadelphia chromosome-positive acute lymphoblastic leukemia receiving tyrosine kinase inhibitors therapy?: A systematic review and meta-analysis
Source: PLoS One. 2021 Jun 28;16(6):e0253896. doi: 10.1371/journal.pone.0253896 (PMC8238225; doi:10.1371/journal.pone.0253896)
Supplement: S1 File — (DOCX) [file pone.0253896.s001.docx]

**S1 File. Searching strategy.**

**EMBASE Database**

1. ‘acute lymphoblastic leukemia’/exp OR ‘acute lymphoblastic leukemia’
2. ‘philadelphia chromosome positive acute lymphoblastic leukemia’/exp OR ‘philadelphia chromosome positive acute lymphoblastic leukemia’
3. ‘hematopoietic stem cell transplantation’/exp OR ‘hematopoietic stem cell transplantation’
4. ‘allogeneic hematopoietic stem cell transplantation’/exp OR ‘allogeneic hematopoietic stem cell transplantation’
5. ‘allogeneic stem cell transplantation’/exp OR ‘allogeneic stem cell transplantation’
6. ‘matched related donor’/exp OR ‘matched related donor’
7. ‘matched sibling donor’/exp OR ‘matched sibling donor’
8. ‘matched unrelated donor’/exp OR ‘matched unrelated donor’
9. ‘haploidentical hematopoietic stem cell transplantation’/exp OR ‘haploidentical hematopoietic stem cell transplantation’
10. ‘haploidentical hematopoietic cell transplantation’/exp OR ‘haploidentical hematopoietic cell transplantation’
11. ‘haploidentical stem cell transplantation’/exp OR ‘haploidentical stem cell transplantation’
12. ‘haploidentical transplantation’/exp OR ‘haploidentical transplantation’
13. ‘haploidentical donor’/exp OR ‘haploidentical donor’
14. ‘unrelated donor’/exp OR ‘unrelated donor’
15. ‘autologous hematopoietic stem cell transplantation’/exp OR ‘autologous hematopoietic stem cell transplantation’
16. ‘autologous stem cell transplantation’/exp OR ‘autologous stem cell transplantation’
17. #1 OR #2
18. #3 OR #4 OR #5 OR #6 OR #7 OR #8 OR #9 OR #10 OR #11 OR #12 OR #13 OR #14 OR #15 OR #16
19. #17 AND #18

**Ovid MEDLINE Database**

1. acute lymphoblastic leukemia.mp. or exp Precursor Cell Lymphoblastic Leukemia-Lymphoma/
2. philadelphia chromosome positive acute lymphoblastic leukemia.mp.
3. exp Transplantation, Homologous/ or exp Hematopoietic Stem Cell Transplantation/ or allogenic hematopoietic stem cell transplantation.mp.
4. stem cell transplantation.mp. or exp Stem Cell Transplantation/
5. allogenic stem cell transplantation.mp.
6. matched related donor.mp.
7. matched sibling donor.mp.
8. matched unrelated donor.mp. or exp Unrelated Donors/
9. haploidentical transplantation.mp. or exp Transplantation, Haploidentical/
10. haploidentical hematopoietic stem cell transplantation.mp.
11. haploidentical donor.mp.
12. autologous transplantation.mp. or exp Transplantation, Autologous/
13. autologous hematopoietic stem cell transplantation.mp.
14. 1 or 2
15. 3 or 4 or 5 or 6 or 7 or 8 or 9 or 10 or 11 or 12 or 13
16. 14 and 15
