# Supplementary material for: Is stem cell transplantation still needed for adult Philadelphia chromosome-positive acute lymphoblastic leukemia receiving tyrosine kinase inhibitors therapy?: A systematic review and meta-analysis
Source: PLoS One. 2021 Jun 28;16(6):e0253896. doi: 10.1371/journal.pone.0253896 (PMC8238225; doi:10.1371/journal.pone.0253896)
Supplement: S1 Table — (DOCX) [file pone.0253896.s003.docx]

|  | **Bassan et al. [17]** | **Li et al. [27]** | **Pfeifer et al. [28]** | **Konopacki et al. [29]** | **Tanguy-Schmidt et al. [15]** | **Fielding et al. [9]** | **Daver et al. [23]** |
| --- | --- | --- | --- | --- | --- | --- | --- |
| Selection | 4 | 3 | 0 | 3 | 4 | 4 | 3 |
| Representativeness of the exposed cohort | Truly representative of the average Ph^+^ ALL patients in the community | Truly representative of the average Ph^+^ ALL patients in the community | No description of the derivation of the cohort | Truly representative of the average Ph^+^ ALL patients in the community | Truly representative of the average Ph^+^ ALL patients in the community | Truly representative of the average Ph^+^ ALL patients in the community | Truly representative of the average Ph^+^ ALL patients in the community |
| Selection of the non-exposed cohort | Drawn from the same community as the exposed cohort | Drawn from the same community as the exposed cohort | No description of the derivation of the non exposed cohort | Drawn from the same community as the exposed cohort | Drawn from the same community as the exposed cohort | Drawn from the same community as the exposed cohort | Drawn from the same community as the exposed cohort |
| Ascertainment of exposure | Secure record | Secure record | No description | Secure record | Secure record | Secure record | Secure record |
| Demonstration that outcome of interest was not present at start of study | Yes | No | No | No | Yes | Yes | No |
|  | | | | | | | |
| Comparability | 2 | 0 | 1 | 1 | 2 | 2 | 1 |
| Study controls for “all patients receiving TKI” | Yes | No | Yes | Yes | Yes | Yes | Yes |
| Study controls for any additional factor | Yes (newly-diagnosed Ph^+^ ALL) | No | No | No | Yes (newly-diagnosed Ph^+^ ALL) | Yes (newly-diagnosed Ph^+^ ALL) | No |
|  | | | | | | | |
| Outcome | 3 | 3 | 1 | 3 | 3 | 3 | 3 |
| Assessment of outcome | Record linkage | Record linkage | No description | Record linkage | Record linkage | Record linkage | Record linkage |
| Was follow-up long enough for outcomes to occur | Yes | Yes | Yes | Yes | Yes | Yes | Yes |
| Adequacy of follow-up of cohorts | Complete follow-up – all subjects accounted for | Complete follow-up – all subjects accounted for | No statement | Complete follow-up – all subjects accounted for | Complete follow-up – all subjects accounted for | Subjects lost to follow up unlikely to introduce bias | Complete follow-up – all subjects accounted for |

**S1 Table. Individual component of the study quality assessment.**

|  | **Ravandi et al. [24]** | **Sun et al. [30]** | **Togasaki et al. [31]** | **Kanfar et al. [32]** | **Kuang et al. [33]** | **Fujisawa et al. [34]** | **Kozlowski et al. [10]** |
| --- | --- | --- | --- | --- | --- | --- | --- |
| Selection | 4 | 4 | 3 | 3 | 4 | 4 | 3 |
| Representativeness of the exposed cohort | Truly representative of the average Ph^+^ ALL patients in the community | Truly representative of the average Ph^+^ ALL patients in the community | Truly representative of the average Ph^+^ ALL patients in the community | Truly representative of the average Ph^+^ ALL patients in the community | Truly representative of the average Ph^+^ ALL patients in the community | Truly representative of the average Ph^+^ ALL patients in the community | Truly representative of the average Ph^+^ ALL patients in the community |
| Selection of the non-exposed cohort | Drawn from the same community as the exposed cohort | Drawn from the same community as the exposed cohort | Drawn from the same community as the exposed cohort | Drawn from the same community as the exposed cohort | Drawn from the same community as the exposed cohort | Drawn from the same community as the exposed cohort | Drawn from the same community as the exposed cohort |
| Ascertainment of exposure | Secure record | Secure record | Secure record | Secure record | Secure record | Secure record | Secure record |
| Demonstration that outcome of interest was not present at start of study | Yes | Yes | No | No | Yes | Yes | No |
|  | | | | | | | |
| Comparability | 2 | 1 | 1 | 1 | 2 | 2 | 2 |
| Study controls for “all patients receiving TKI” | Yes | No | Yes | Yes | Yes | Yes | Yes |
| Study controls for any additional factor | Yes (CR1) | Yes (CR1) | No | No | Yes (CR1) | Yes (CR1) | Yes (CR1) |
|  | | | | | | | |
| Outcome | 3 | 3 | 3 | 3 | 3 | 3 | 2 |
| Assessment of outcome | Record linkage | Record linkage | Record linkage | Record linkage | Record linkage | Record linkage | Record linkage |
| Was follow-up long enough for outcomes to occur | Yes | Yes | Yes | Yes | Yes | Yes | Yes |
| Adequacy of follow-up of cohorts | Complete follow-up – all subjects accounted for | Complete follow-up – all subjects accounted for | Complete follow-up – all subjects accounted for | Complete follow-up – all subjects accounted for | Subjects lost to follow-up unlikely to introduce bias | Complete follow-up – all subjects accounted for | No statement |

|  | **Hatta et al. [11]** | **Jabbour et al. [26]** | **Agrawal et al. [12]** | **Chang et al. [16]** | **Ghobadi et al. [36]** | **Wang et al. [37]** | **Wetzler et al. [22]** |
| --- | --- | --- | --- | --- | --- | --- | --- |
| Selection | 4 | 4 | 3 | 3 | 4 | 3 | 3 |
| Representativeness of the exposed cohort | Truly representative of the average Ph^+^ ALL patients in the community | Truly representative of the average Ph^+^ ALL patients in the community | Truly representative of the average Ph^+^ ALL patients in the community | Truly representative of the average Ph^+^ ALL patients in the community | Truly representative of the average Ph^+^ ALL patients in the community | Truly representative of the average Ph^+^ ALL patients in the community | Truly representative of the average Ph^+^ ALL patients in the community |
| Selection of the non-exposed cohort | Drawn from the same community as the exposed cohort | Drawn from the same community as the exposed cohort | Drawn from the same community as the exposed cohort | Drawn from the same community as the exposed cohort | Drawn from the same community as the exposed cohort | Drawn from the same community as the exposed cohort | Drawn from the same community as the exposed cohort |
| Ascertainment of exposure | Secure record | Secure record | Secure record | Secure record | Secure record | Secure record | Secure record |
| Demonstration that outcome of interest was not present at start of study | Yes | Yes | No | No | Yes | No | No |
|  | | | | | | | |
| Comparability | 2 | 2 | 1 | 2 | 2 | 2 | 1 |
| Study controls for “all patients receiving TKI” | Yes | Yes | Yes | Yes | Yes | Yes | Yes |
| Study controls for any additional factor | Yes (CR1) | Yes (CR1) | No | Yes (CR1) | Yes (CR1) | Yes (CR1) | No |
|  | | | | | | | |
| Outcome | 3 | 3 | 3 | 3 | 3 | 3 | 3 |
| Assessment of outcome | Record linkage | Record linkage | Record linkage | Record linkage | Record linkage | Record linkage | Record linkage |
| Was follow-up long enough for outcomes to occur | Yes | Yes | Yes | Yes | Yes | Yes | Yes |
| Adequacy of follow-up of cohorts | Subjects lost to follow-up unlikely to introduce bias | Complete follow-up – all subjects accounted for | Subjects lost to follow-up unlikely to introduce bias | Complete follow-up – all subjects accounted for | Complete follow-up – all subjects accounted for | Complete follow-up – all subjects accounted for | Complete follow-up – all subjects accounted for |

|  | **Chalandon et al. [13]** | **Tan et al. [25]** | **Liu et al. [35]** | **Giebel et al. [14]** | **Lyu et al. [38]** |
| --- | --- | --- | --- | --- | --- |
| Selection | 4 | 0 | 0 | 4 | 3 |
| Representativeness of the exposed cohort | Truly representative of the average Ph^+^ ALL patients in the community | No description of the derivation of the cohort | No description of the derivation of the cohort | Truly representative of the average Ph^+^ ALL patients in the community | Truly representative of the average Ph^+^ ALL patients in the community |
| Selection of the non-exposed cohort | Drawn from the same community as the exposed cohort | No description of the derivation of the non exposed cohort | No description of the derivation of the non exposed cohort | Drawn from the same community as the exposed cohort | Drawn from the same community as the exposed cohort |
| Ascertainment of exposure | Secure record | No description | No description | Secure record | Secure record |
| Demonstration that outcome of interest was not present at start of study | Yes | No | No | Yes | No |
|  | | | | | |
| Comparability | 2 | 2 | 1 | 1 | 1 |
| Study controls for “all patients receiving TKI” | Yes | Yes | Yes | No | Yes |
| Study controls for any additional factor | Yes (newly-diagnosed Ph^+^ ALL) | Yes (CR1) | No | Yes (CR1) | No |
|  | | | | | |
| Outcome | 3 | 1 | 1 | 3 | 3 |
| Assessment of outcome | Record linkage | No description | No description | Record linkage | Record linkage |
| Was follow-up long enough for outcomes to occur | Yes | Yes | Yes | Yes | Yes |
| Adequacy of follow-up of cohorts | Subjects lost to follow-up unlikely to introduce bias | No statement | No statement | Complete follow-up – all subjects accounted for | Complete follow-up – all subjects accounted for |
