# Supplementary figures and images for: Is stem cell transplantation still needed for adult Philadelphia chromosome-positive acute lymphoblastic leukemia receiving tyrosine kinase inhibitors therapy?: A systematic review and meta-analysis
Source: PLoS One. 2021 Jun 28;16(6):e0253896. doi: 10.1371/journal.pone.0253896 (PMC8238225; doi:10.1371/journal.pone.0253896)

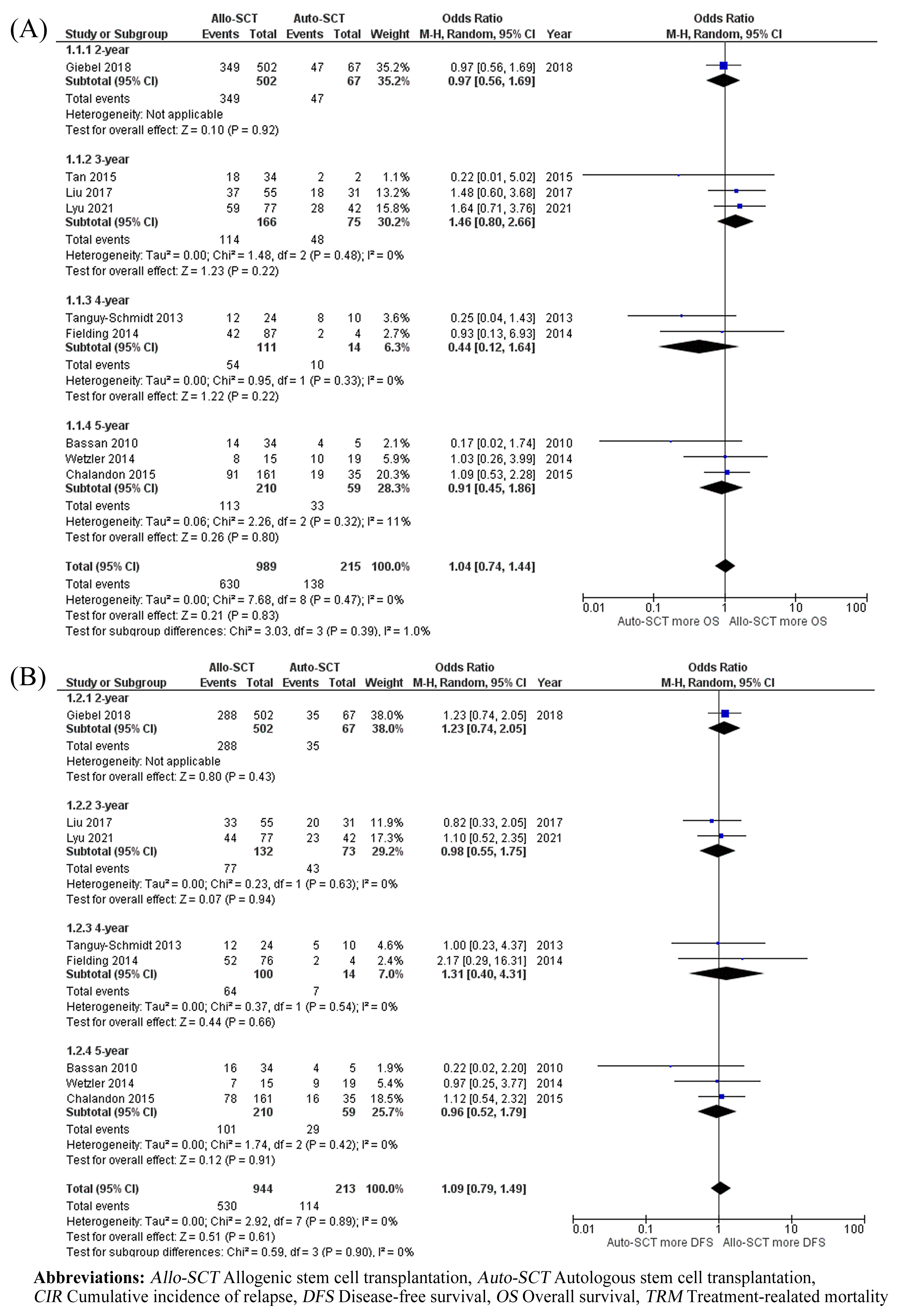

Supplement: S1 Fig — (A) OS rate between allo-HSCT versus auto-HSCT (B) DFS rate between allo-HSCT versus auto-HSCT. (TIF) [file pone.0253896.s004.tif]

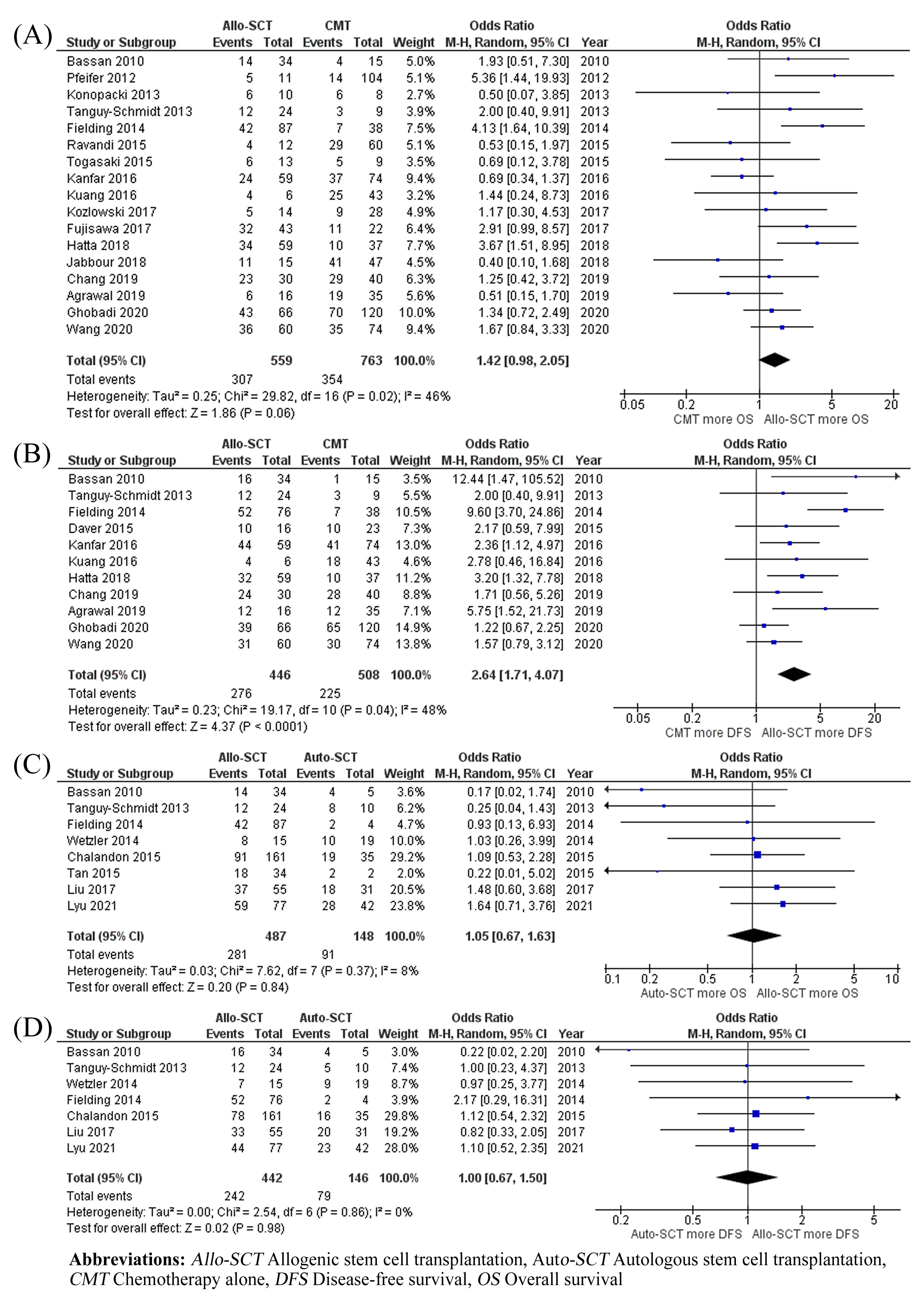

Supplement: S2 Fig — (A) OS rate between allo-HSCT versus CMT only (B) DFS rate between allo-HSCT versus CMT (C) OS rate between allo-HSCT versus auto-HSCT (D) DFS rate between allo-HSCT versus auto-HSCT. (TIF) [file pone.0253896.s005.tif]

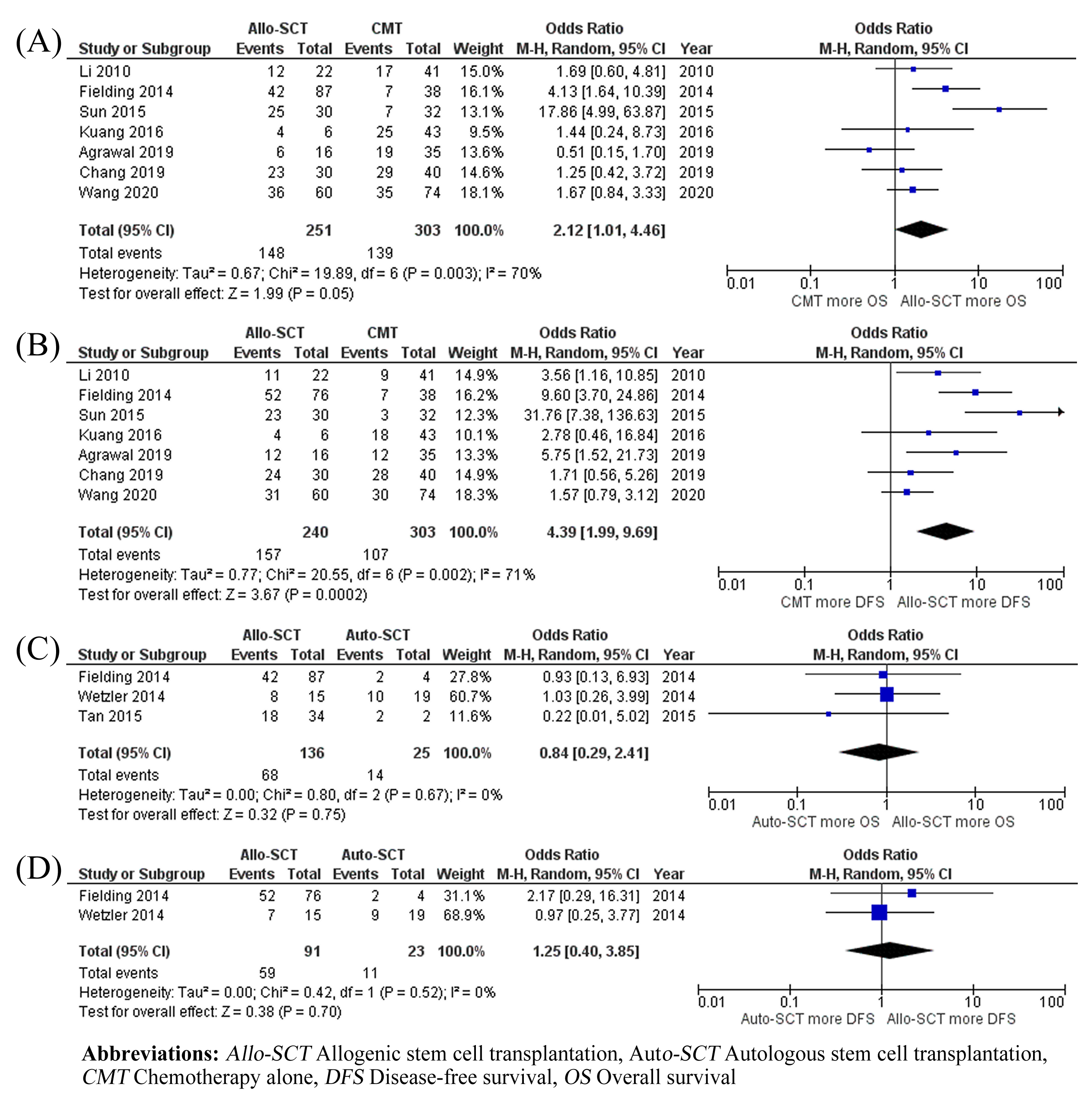

Supplement: S3 Fig — (A) OS rate between allo-HSCT versus CMT only (B) DFS rate between allo-HSCT versus CMT (C) OS rate between allo-HSCT versus auto-HSCT (D) DFS rate between allo-HSCT versus auto-HSCT. (TIF) [file pone.0253896.s006.tif]

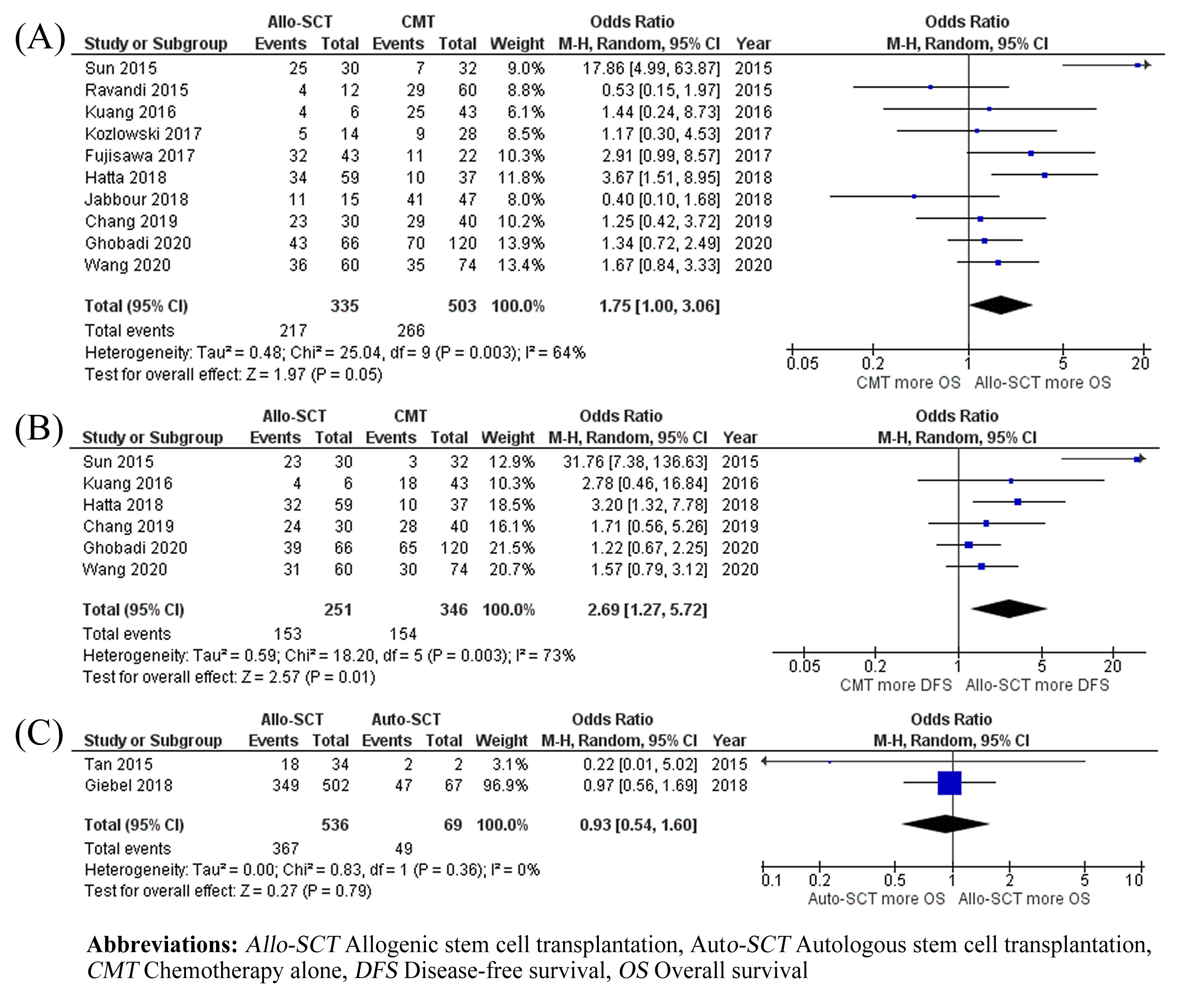

Supplement: S4 Fig — (A) OS rate between allo-HSCT versus CMT only (B) DFS rate between allo-HSCT versus CMT (C) OS rate between allo-HSCT versus auto-HSCT. (TIF) [file pone.0253896.s007.tif]

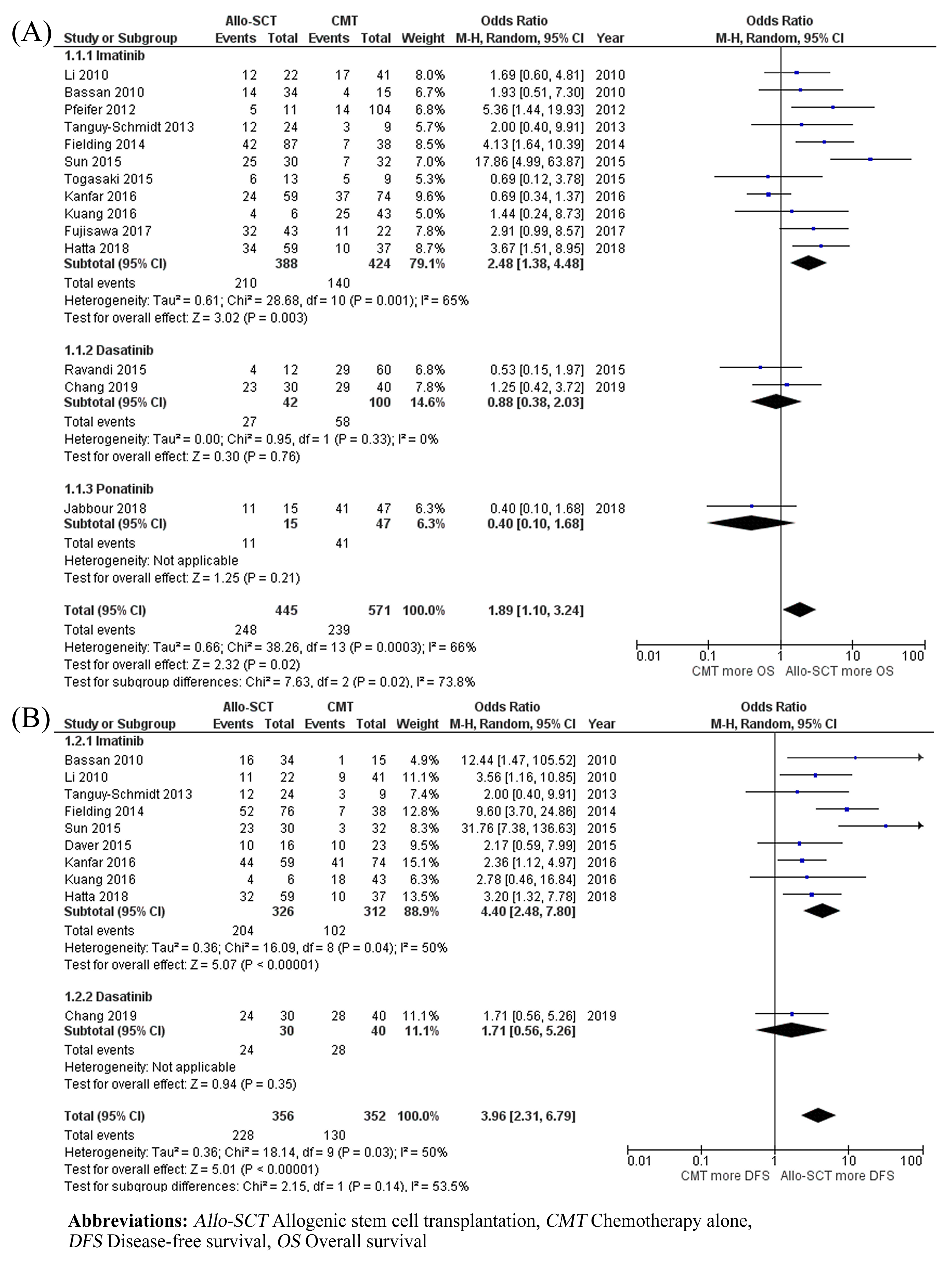

Supplement: S5 Fig — (A) OS rate between allo-HSCT versus CMT only (B) DFS rate between allo-HSCT versus CMT. (TIF) [file pone.0253896.s008.tif]
